# Nomogram Incorporating CD44v6 and Clinicopathological Factors to Predict Lymph Node Metastasis for Early Gastric Cancer

**DOI:** 10.1371/journal.pone.0159424

**Published:** 2016-08-02

**Authors:** Bang Wool Eom, Jungnam Joo, Boram Park, Min Jung Jo, Seung Ho Choi, Soo-Jeong Cho, Keun Won Ryu, Young-Woo Kim, Myeong-Cherl Kook

**Affiliations:** 1 Department of Medicine, Yonsei University Graduate School of Medicine, Seoul, Republic of Korea; 2 Gastric Cancer Branch, Research Institute & Hospital, National Cancer Center, Goyang, Gyeonggi-do, Republic of Korea; 3 Cancer Biostatistics Branch, Research Institute for National Cancer Control & evaluation, National Cancer Center, Goyang, Gyeonggi-do, Republic of Korea; Sapporo Medical University, JAPAN

## Abstract

**Background:**

Treatment strategy for early gastric cancer depends on the probability of lymph node metastasis. The aim of this study is to develop a nomogram predicting lymph node metastasis in early gastric cancer using clinicopathological factors and biomarkers.

**Methods:**

A literature review was performed to identify biomarkers related to lymph node metastasis in gastric cancer. Seven markers were selected and immunohistochemistry was performed in 336 early gastric cancer tissues. Based on the multivariable analysis, a prediction model including clinicopatholgical factors and biomarkers was developed, and benefit of adding biomarkers was evaluated using the area under the receiver operating curve and net reclassification improvement. Functional study in gastric cancer cell line was performed to evaluate mechanism of biomarker.

**Results:**

Of the seven biomarkers studied, α1 catenin and CD44v6 were significantly associated with lymph node metastasis. A conventional prediction model, including tumor size, histological type, lymphatic blood vessel invasion, and depth of invasion, was developed. Then, a new prediction model including both clinicopathological factors and CD44v6 was developed. Net reclassification improvement analysis revealed a significant improvement of predictive performance by the addition of CD44v6, and a similar result was shown in the internal validation using bootstrapping. Prediction nomograms were then constructed based on these models. In the functional study, CD44v6 was revealed to affect cell proliferation, migration and invasion.

**Conclusions:**

Overexpression of CD44v6 was a significant predictor of lymph node metastasis in early gastric cancer. The prediction nomograms incorporating CD44v6 can be useful to determine treatment plans in patients with early gastric cancer.

## Introduction

Treatment strategy for early gastric cancer depends on the probability of lymph node metastasis. Generally, radical gastrectomy with lymph node dissection is the treatment of choice for localized gastric cancer. However, endoscopic resection is considered for tumors with a very low risk of lymph node metastasis. [1, 2] Probability of lymph node metastasis is estimated by several clinicopatholgoical factors such as tumor size and histological type, and endoscopic resection is performed for the tumor meeting the indications for endoscopic resection. [3] However, recent studies reported considerable incidences of lymph node metastasis in tumors meeting the expanded criteria, and oncological safety of endoscopic treatment is still debated. Thus, a new marker with high predictability of lymph node metastasis is required. [4–6]

To date, numerous biomarkers have been demonstrated to be associated with lymph node metastasis in gastric cancer. However, most studies have been undertaken in cases of advanced gastric cancer tissues, and few studies have reported significant association between biomarkers and lymph node metastasis in early gastric cancer tissues. [7–10] The aim of this study is to identify biomarkers related to lymph node metastasis and to develop a prediction nomogram for lymph node metastasis in early gastric cancer using clinicopathological factors and biomarkers.

## Material and methods

### Literature search

A literature review was performed to identify immunohistochemical markers predicting lymph node metastasis in gastric cancer. The database PubMed was searched using the following combination of terms: “lymph node metastasis”, “gastric cancer”, and “predict OR prediction”. A total of 166 studies published before 22 October, 2013 was evaluated, and duplicate or non-English publications were excluded. We also excluded 9 studies that did not include any biomarkers, 13 studies for serum markers, 8 gene studies, and 2 other studies using cell lines and mouse models. Of the remaining 134 studies, we selected 3 markers examined in early gastric cancer tissue (E-cadherin, α1 catenin, and p53) and 4 markers identified to be significant in multivariable analyses (EZH2 (enhancer of zeste homolog 2), Annexin II, CD44v6, and PRL-3 (phosphatase regenerating liver)).

### Patients and tissue samples

Early gastric cancer tissues were obtained from 336 patients who underwent D2 gastrectomies at the National Cancer Center, Korea between January and September 2006. Written informed consent for use of the surgical specimens was obtained from all patients preoperatively, and the Institutional Review Board (IRB) of the National Cancer Center, Korea approved this study (No. NCCNCS 13–822).

### Immunohistochemistry

A tissue microarray (TMA) was constructed from the paraffin-embedded blocks of 336 early gastric cancer tissues using a tissue array device (Beecher Instruments Inc., Sun Prairie, WI). A core tissue 2 mm in diameter was taken from the marked tumor area and arranged in recipient paraffin blocks. The TMA blocks were sectioned at 4 μm, and the sections were mounted on precoated glass slides and deparaffinized.

The antigens were retrieved with heat treatment for 30 minutes in pH 8.0 Tris-EDTA buffer (CC1, Ventana medical systems, Tucson, AZ) at 95°C. Endogenous peroxidases were blocked with 3% H_2_O_2_ for 10 minutes at room temperature. Nonspecific binding blocking was performed with a ready-to-use protein blocker solution (Ventana medical systems, Tucson, AZ) for 20 minutes at RT.

The following primary antibodies were used: E-cadherin (1:250, 61082, BD Biosciences, San Jose, CA), alpha 1 Catenin (1:400, ab49105, Abcam, Cambridge, MA), p53 (1:500, ab80644, Abcam, Cambridge, MA), KMT6/EZH2 (phospho T487) (1:200, ab109398, Abcam, Cambridge, MA), Annexin A2 (1:1000, ab41803, Abcam, Cambridge, MA), CD44v6 (1:125, BBA13, R&D Systems, Minneapolis, MN), and PRL (1:250, MAB32191, R&D Systems, Minneapolis, MN). The sections were incubated with primary antibodies for 32 minutes in 42°C and then labeled with HPR multimer labeled secondary antibody (ultraView Universal DAB detection kit, Ventana medical systems, Tucson, AZ) for 20 minutes at room temperature and stained for 8 minutes, followed by hematoxylin counterstaining.

### Assessment of staining

The staining intensity was defined as follows: 0, no signal or positive tumor cells <10%; 1, visible but weak signal in x100 power; 2, clear signal in x100 power; 3, apparent in x40 power. The percentage of positive tumor cells was measured as follows: 0, absent; 1, <10%; 2, 10–50%; 3, 50–90%; 4, >90%. The score was produced by multiplying the intensity and the percentage. E-cadherin, α1 catenin, and EZH were detected with intensity 2 in normal foveolar epithelial cells, and abnormal immunoreactivity was defined as a moderate decrease of the signal (score ≤4). In contrast, p53 and CD44v6 were absent or weak positive in normal foveolar epithelial cells, and positive immunoreactivity of p53 and CD44v6 was defined as an apparent detection of the signal (score ≥4).

### Clinicopathological characteristics

The histological classification was performed according to the World Health Organization classification, and histological types were categorized according to the recent Japanese treatment guidelines. [1, 11] Lymphatic blood vessel invasion (LBVI) was defined as tumor cells spreading through the lymphatic or venous vessels, and was categorized as “not identifies” or “present”. Ulceration was not determined by pathological findings, but by endoscopy. When a marked ulcer was present or the tumor was EGC type 0-III (excavated) in endoscopic evaluation, it was considered an ulceration. [12] The cancers were staged in accordance with the 7^th^ American Joint Committee on Cancer (AJCC) tumor-node-metastasis (TNM) classification. [13]

### Cell culture and transfection

Human gastric cancer cell lines obtained from the Korea Cell Line Bank. Cells were maintained in RPMI 1640 (Gibco, life technologies, Carlsbad, CA) containing 10% fetal bovine serum, 1% Antibiotic-Antimycotic, and humidified atmosphere of 5% CO2 / 95% air maintained at 37°C.

SiRNA transfection in YCC-2 were performed using the Lipofectamine RNAiMAX reagent (Invitrogen, Carlsbad, CA) according to the manufacturer’s protocol using 5 nM of CD44 standard (CD44s) siRNA: (5′-AGCTCTGAGCATCGGATTT-3′; 5′-TGGCTGATCATCTTGGCAT-3′; Gene Pharma, Shanghai, China), 5 nM of CD44v6-10 siRNA: (5′-GCAACTCCTAGTAGTACAAdTdT-3′ and 5′-TGAGGGATATCGCCAAACAdTdT-3′; Gene Pharma, Shanghai, China) and negative control siRNA: 5′-UUCUCCGAACGUGUCACGUTT-3′; 5′-ACGUGACACGUUCGGAGAATT-3′; Gene Pharma, Shanghai, China). The lentivirus- mediating CD44s and CD44v6-10 plasmids and control vector were transfected in MKN-28 cells for overexpression. Three days after infection, ampicillin was added to the media at 2ug/ml and cell populations were selected for 2 weeks. Knockdown and overexpression of CD44v6 were evaluated by RT-PCR and western blot.

### Cell proliferation, migration and invasion analysis

Cell proliferation was measured with the MTT assay. YCC-2 and MKN-28 cells were plated in 96-well culture plates (5 × 10^3^ per well), followed by transfection of synthetic CD44s siRNA, CD44v6 siRNA, scRNA, CD44s plasmid, and CD44v6 plasmid. After 48 hours of incubation, the MTT (0.5 mg/ml) (Sigma-Aldrich, St. Louis, MO) was subsequently added to each well (200 μl/well). After 4 hours of additional incubation, the MTT solution was discarded; and 200 μl of DMSO (Amresco, Solon, OH) was added, and the plate was shaken gently. The absorbance was measured on an enzymelinked immunosorbent assay reader at a wavelength of 450 nm.

Trans-filter migration and invasion assays were performed on YCC-2 and MKN-28 cell lines in serum-free RPMI with 8.0-μm pore inserts on a 24-well Transwell (Corning Costar, Lowell, MA). The cell lines were transfected with synthetic CD44s siRNA, CD44v6 siRNA, scRNA, CD44s plasmid, and CD44v6 plasmid for 1 day and then transferred to the upper chamber of the Transwell coated with 0.5 mg/ml collagen type I and a 1:15 dilution of Matrigel (BD Bioscience, San Jose, CA). Migrating and invading cells were quantified after H&E staining. Migration and invasion assays were performed after transfection, as previously described.

### Statistical analysis

All continuous variables were expressed as the means ± standard deviations (SD), and the categorical variables were presented as portions. Continuous variables were analyzed using the independent sample t-test, and differences in proportions were tested using the two-tailed chi-square test or Fisher’s exact test.

The binary logistic regression model was used to estimate odds ratio of lymph node metastasis for each of the risk factors. A backward selection method with a type I error criterion of 0.1 was used in the multivariable model. Two types of models using conventional clinicopathological factors were developed: one with and the other without LBVI. Then, the biomarkers were added to the conventional prediction models. The benefits of adding a biomarker to the conventional model were evaluated using the area under the receiver operating curve (AUC), and net reclassification improvement (NRI). [14]

A bootstrap approach was employed for internal validation. [15] Random samples of the same sample size were derived from the original data and formed bootstrap samples. The same technique based on backward variable selection was applied on the bootstrap samples to develop a bootstrap-prediction model. The difference in performance between the bootstrap-prediction model and the original dataset represents the bias indicating over-fitting. This bootstrap resampling procedure was repeated 2000 times to obtain the average bias, which was corrected to provide a bias-corrected estimate for performance measures. This bias-corrected measure indicates how well the model will perform on an independent data. Finally, we developed two nomograms predicting lymph node metastasis in early gastric cancer for clinical usage.

All data were analyzed using SAS software version 9.1.3 (SAS Institute, Cary, NC) and R (version 13.0.2), and statistical significance was accepted for p values < 0.05.

## Results

### E-cadherin, α1 catenin, p53, EZH, and CD44v6 expression

**Fig 1:**
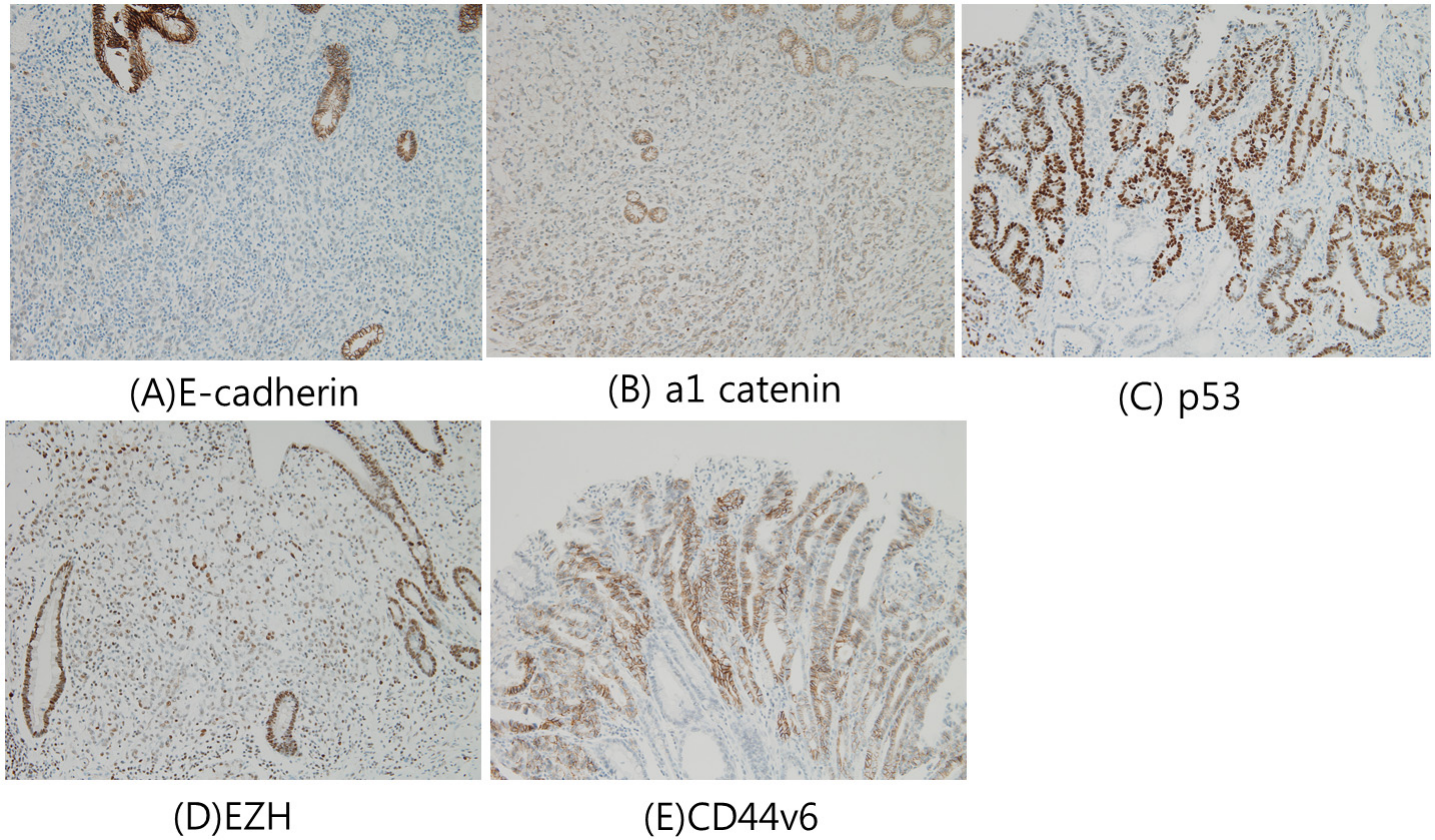
Immunochemical staining of selected biomarkers in early gastric cancer tissues. (A) E-cadherin (B) a1 catenin (C) p53 (D) EZH (E) CD44v6.

Seven markers identified from the literature review were immunostained (Fig 1A–1E). E-cadherin was expressed in the foveolar epithelial cells and glandular cells, and 14.6% of cancers had low expression of E-cadherin. The marker α1 catenin was also expressed in gastric and intestinal metaplastic epithelial cells, and its expression was low in 25.6% of cancers. The marker p53 was rarely expressed in the neck area of gastric pits or the base of intestinal metaplastic glands, and expression of p53 was observed in 33.9% of cancers. EZH was expressed in the neck area of foveolar epithelial cells, and its expression was lower in 19.3% of cancers. CD44v6 was rarely expressed in the neck area of gastric pits, and was expressed in 9.2% of cancers. The remaining 2 markers, Annexin A2 and PRL-3, had little discrimination and could not be evaluated. Most cells of various types in normal tissues and all tumor cells were stained with uniform intensity for Annexin A2 and PRL-3.

### Clinicopathological factors and immunostaining results according to lymph node status

The distribution of pathological stage were as follows; stage IA (n = 312), IB (n = 23), and IIB (n = 1, T1N3).

Table 1 shows the clinicopathological characteristics and the immunostaining results for five markers according to lymph node metastasis. The tumor size was larger, and the proportions of undifferentiated type, LBVI, and submucosal invasion were higher in the lymph node positive group than in the lymph node negative group. Of the five markers, only α1 catenin and CD44v6 were significantly different between the two groups (*P* = 0.018 and 0.003, respectively).

**Table 1:** Clinicopathological characteristics and immunostaining results according to lymph node metastasis.

Characteristics	Subgroup	Total (n = 336)	LN (-) (%) (n = 312)	LN (+) (%) (n = 24)	*p*-value
Age (mean±SD)		336	58.37±11.3	55.17±11.42	0.182
Sex	Male	211	196 (62.8)	15 (62.5)	0.975
	Female	125	116 (37.2)	9 (37.5)	
Size (mean±SD)		336	3.09±1.76	4.58±2.49	0.009
Tumor location	Lower third	178	161 (51.6)	17 (70.8)	0.439
	Middle third	116	110 (35.3)	6 (25.0)	
	Upper third	30	29 (9.3)	1 (4.2)	
	Overlapping	12	12 (3.8)	0 (0)	
Histological type	Differentiated	212	203 (65.1)	9 (37.5)	0.007
	Undifferentiated	124	109 (34.9)	15 (62.5)	
LBVI	Not identified	306	290 (92.9)	16 (66.7)	< 0.001
	Present	30	22 (7.1)	8 (33.3)	
Ulceration*	Absent	320	299 (95.8)	21 (87.5)	0.097
	Present	16	13 (4.2)	3 (12.5)	
Depth of invasion	Mucosa	195	191 (61.2)	4 (16.7)	< 0.001
	Submucosa	141	121 (38.8)	20 (83.3)	
E-cadherin	Not reduced	287	266 (82.3)	21 (87.5)	0.999
	Reduced	49	46 (14.7)	3 (12.5)	
α1 catenin	Not reduced	250	237 (76.0)	13 (54.2)	0.018
	Reduced	86	75 (24.0)	11 (45.8)	
p53	Low	222	207 (66.3)	15 (62.5)	0.701
	High	114	105 (33.7)	9 (37.5)	
EZH	Not reduced	271	253 (81.1)	18 (75.0)	0.431
	Reduced	65	59 (18.9)	6 (25.0)	
CD44v6	Low	305	288 (92.3)	17 (70.8)	0.003
	High	31	24 (7.7)	7 (29.2)	

LN, lymph, node; LBVI, lymphatic blood vessel invasion

*Ulceration is endoscopic finding

We also evaluated the clinicopathological characteristics according to the expression of CD44v6, and LBVI and lymph node metastasis were significantly higher in the CD44v6 high group (S1 Table).

### Multivariable analysis of factors related to lymph node metastasis

We performed two types of univariable and multivariable analyses according to the presence of LBVI. (Tables 2 and 3) The model with LBVI can be used to predict lymph node metastasis after endoscopic submucosal dissection (ESD), and the model without LBVI can be used before ESD or operation, because LBVI is identified after pathological examination of ESD or in surgical specimens. In the multivariable analyses using clinicopathological factors (conventional model), tumor size, histological type, LBVI, and depth of invasion were significant predictors for lymph node metastasis. Then, five biomarkers were added to the conventional model, and new models incorporating biomarkers were developed (biomarker model). In the biomarker model, tumor size, histological type, LBVI, depth of invasion, and CD44v6 were independent factors. In particular, CD44v6 had the highest odds ratio in the prediction model with LBVI (odds ratio, 5.75; 95% confidence interval (CI), 1.8 ~ 18.44), and the second highest in the model without LBVI (odds ratio, 6.45; 95% CI, 2.02 ~ 20.56).

**Table 2:** Risk factors for lymph node metastasis. Model including LBVI.

Factors	Subgroup	Univariable analysis		Multivariable analysis (Clinicopathological factors)		Multivariable analysis (Clinicopathological + Biomarkers)	
		Hazard ratio (95% CI)	*P*	Hazard ratio (95% CI)	*P*	Hazard ratio (95% CI)	*P*
Age (mean±SD)		0.98 (0.94–1.01)	0.183				
Sex	Male	1					
	Female	1.01 (0.43–2.39)	0.975				
Tumor size (mean±SD)		1.37 (1.15–1.64)	<0.001	1.33 (1.08–1.63)	0.006	1.33 (1.07–1.65)	0.01
Tumor location	Lower	1	0.452				
	Middle	0.52 (0.20–1.35)	0.178				
	Upper	0.33 (0.04–2.55)	0.327				
	Overlapping	NA	0.999				
Histological type	Differentiated	1		1		1	
	Undifferentiated	3.10 (1.32–7.33)	0.001	2.54 (1.01–6.42)	0.049	3.30 (1.21–8.96)	0.019
LBVI	Not identified	1		1			
	Present	6.59 (2.54–17.09)	<0.001	3.20 (1.10–9.29)	0.033	2.81 (0.94–8.46)	0.066
Ulceration	Absent	1					
	Present	3.29 (0.87–12.44)	0.08				
Depth of invasion	Mucosa	1		1		1	
	Submucosa	7.89 (2.63–23.65)	<0.001	5.45 (1.66–17.89)	0.005	5.48 (1.63–18.43)	0.006
E-cadherin	Not reduced	1					
	Reduced	0.83 (0.24–2.88)	0.765				
α1 catenin	Not reduced	1					
	Reduced	2.67 (1.15–6.22)	0.022				
p53	Low	1					
	High	1.18 (0.50–2.79)	0.702				
EZH	Not reduced	1					
	Reduced	1.43 (0.54–3.76)	0.469				
CD44v6	Low	1				1	
	High	4.94 (1.87–13.08)	0.001			5.75 (1.79–18.44)	0.003

LBVI, lymphatic blood vessel invasion; CI, confidence interval; NA, not applicable

**Table 3:** Risk factors for lymph node metastasis. Models excluding LBVI.

Factors	Subgroup	Multivariable analysis (Clinicopathological factors)		Multivariable analysis (Clinicopathological factors + Biomarkers)	
		Hazard ratio (95% CI)	*P*	Hazard ratio (95% CI)	*P*
Age (mean±SD)					
Sex	Male				
	Female				
Tumor size (mean±SD)		1.35 (1.10–1.65)	0.004	1.34 (1.09–1.66)	0.006
Tumor location	Lower				
	Middle				
	Upper				
	Overlapping				
Histological type	Differentiated	1		1	
	Undifferentiated	2.32 (0.94–5.72)	0.068	3.09 (1.16–8.23)	0.025
Ulceration	Absent				
	Present				
Depth of invasion	Mucosa	1		1	
	Submucosa	7.52 (2.42–23.36)	0.001	7.52 (2.37–23.83)	<0.001
E-cadherin	Not reduced				
	Reduced				
α1 catenin	Not reduced				
	Reduced				
p53	Low				
	High				
EZH	Not reduced				
	Reduced				
CD44v6	Low			1	
	High			6.45 (2.02–20.56)	0.002

LBVI, lymphatic blood vessel invasion; CI, confidence interval

### Performance of predictive models including CD44v6

In the model with LBVI, the AUC slightly increased, from 0.851 (95% CI, 0.784~0.918) to 0.865 (95% CI, 0.804~0.926). Similarly, an increase in the AUC from 0.831 (95% CI, 0.766~0.895) to 0.853 was observed by adding CD44v6 in the model without LBVI. After upward bias correction using bootstrapping, the AUCs were still high, with a bias-corrected AUC of 0.806 in the biomarker model with LBVI and 0.80 in the biomarker model without LBVI

Then, the benefit of adding CD44v6 to the conventional model was tested by evaluating NRI. A P-value of 0.043 was calculated for both models, which suggests that significant improvements in reclassification were induced by adding CD44v6.

### Nomogram

**Fig 2:**
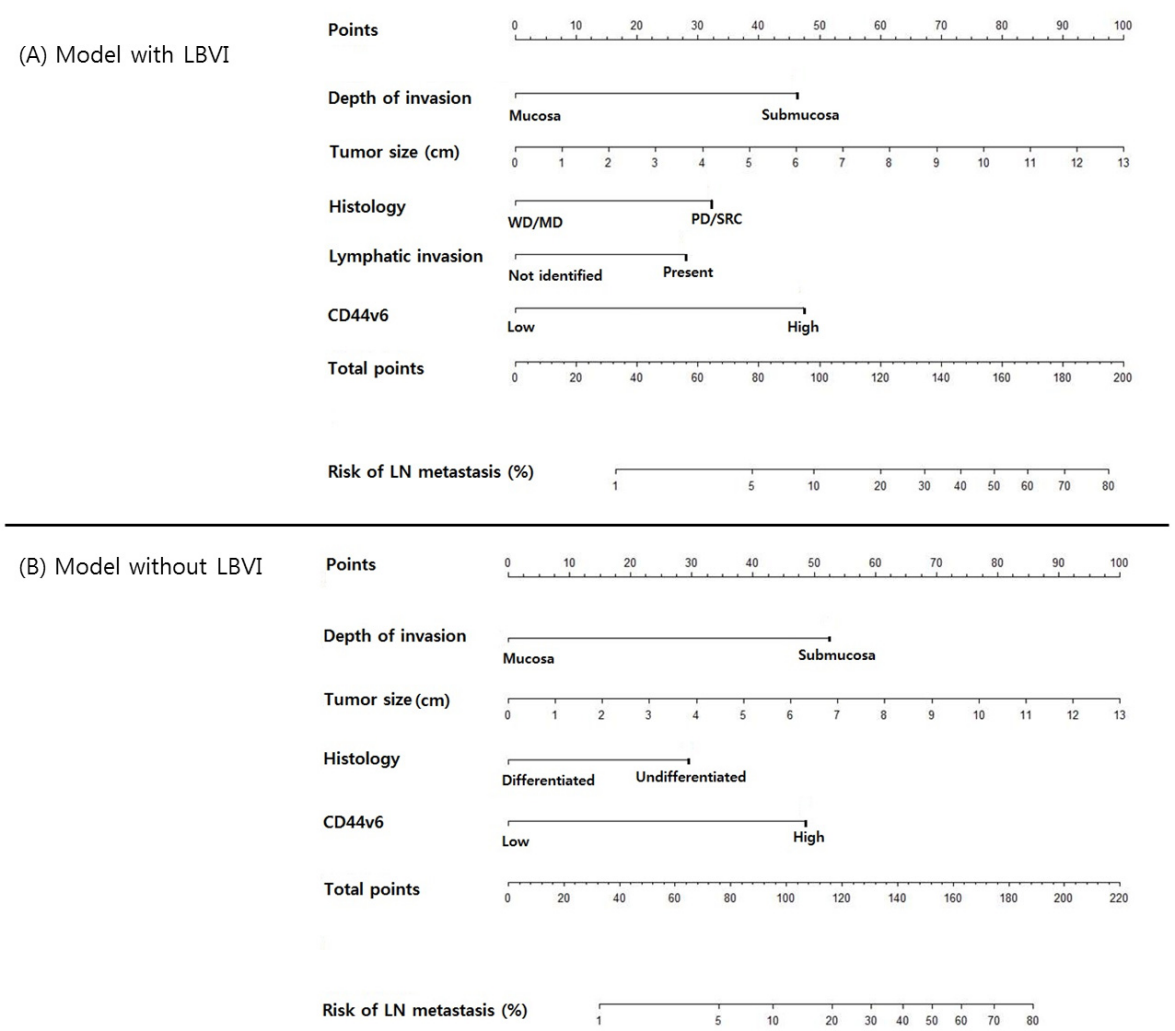
Two nomograms predicting the probability of lymph node metastasis in early gastric cancer. (A) including LBVI (B) excluding LBVI.

Finally, we developed two nomograms predicting lymph node metastasis in early gastric cancer based on biomarker models (Fig 2). Each predicting factor has a specific point, and the sum of the points indicates the probability of lymph node metastasis. For example, a 3 cm, well-differentiated submucosal cancer without lymphatic invasion was reported after ESD, and the expression of CD44v6 was high in immunohistochemistry. In this case, the total point equals 117 and the probability of lymph node metastasis is 18%. This calculated value could be used in decision making for treatment plans and patient counseling.

### Function of CD44v6 in gastric cancer cell line

**Fig 3:**
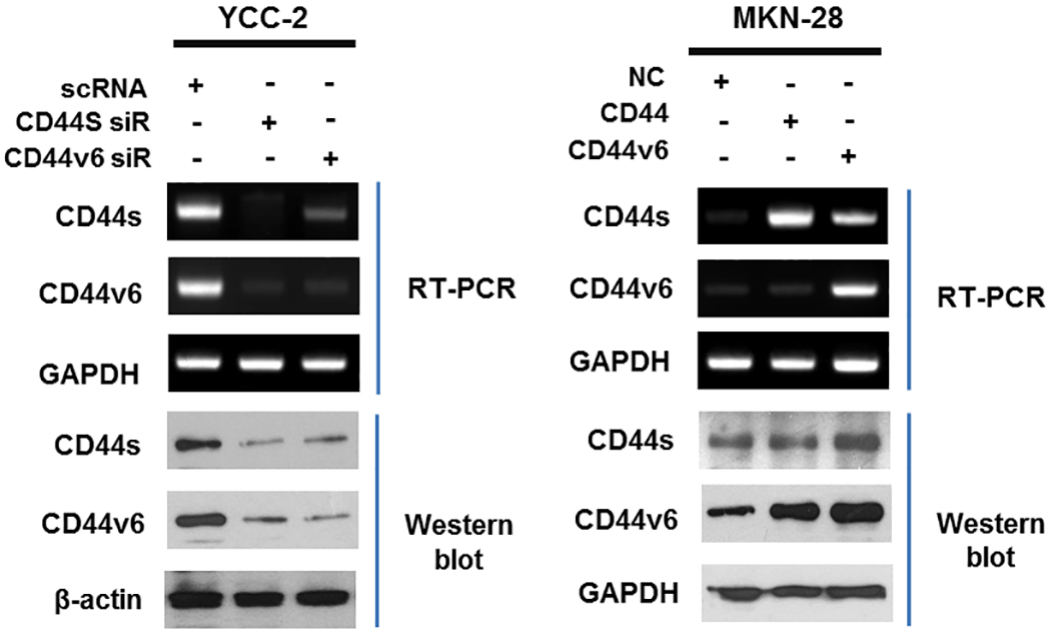
Knockdown of CD44v6 with siRNA transfection in YCC-2 cell line and overexpression of CD44v6 with plasmid transfection in MKN-28 cell line.

CD44v6 expressions were evaluated by RT-PCR and Western blot after knockdown with siRNA transfection in YCC-2 cell line and after over-expressive plasmid transfection in MKN-28 cell line (Fig 3).

**Fig 4:**
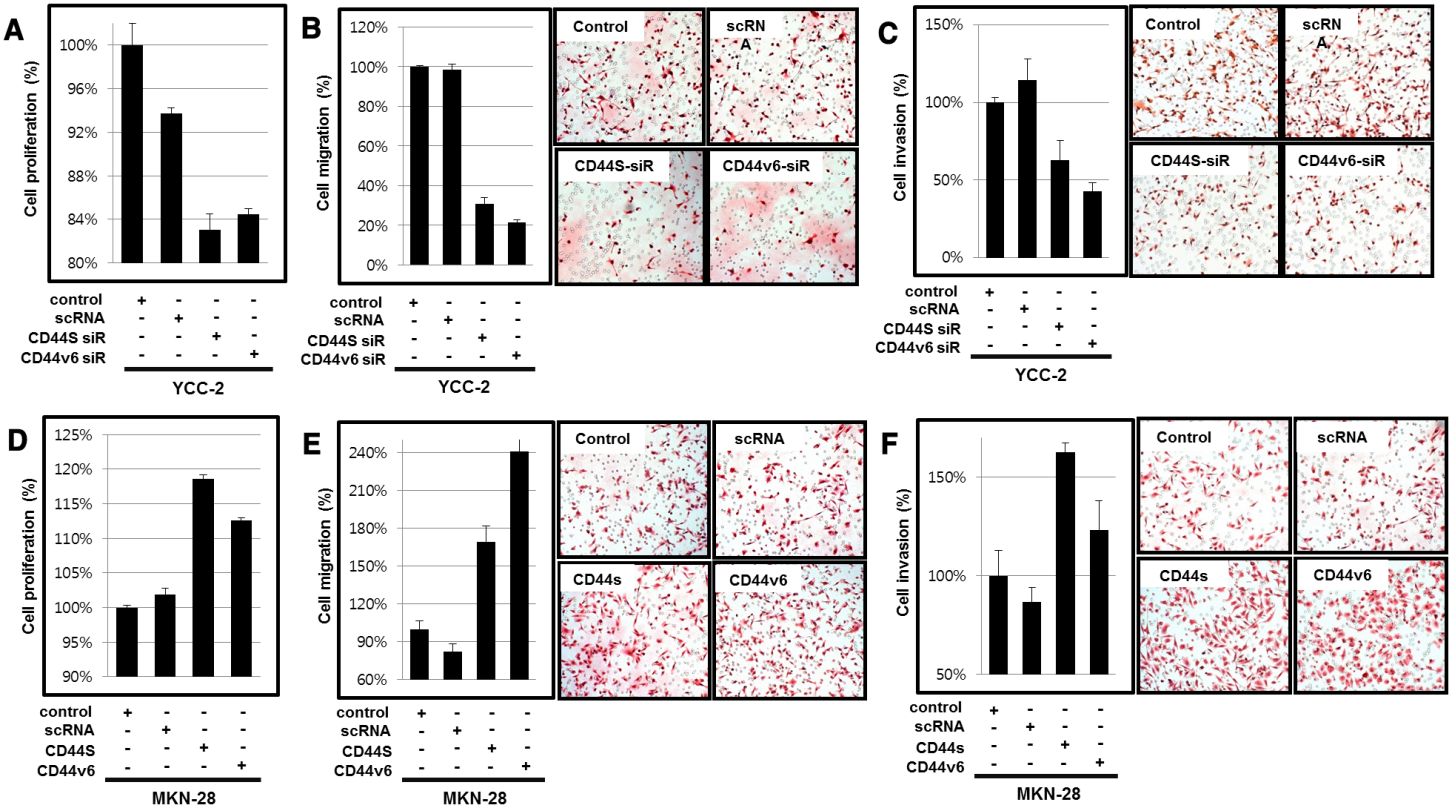
(A) Proliferation analysis (B) migration analysis (C) invasion analysis after knockdown of CD44v6 in YCC-2 cell. (D) Proliferation analysis (E) migration analysis (F) invasion analysis after overexpression of CD44v6 in MKN-28 cell line.

We performed cell proliferation, migration and invasion analyses after knockdown and overexpression to investigate the function of CD44v6. Knockdown of CD44v6 inhibited cell proliferation, migration and invasion (Fig 4A–4C). On the other hands, overexpression of CD44v6 promoted cell proliferation, migration and invasion (Fig 4E and 4F).

## Discussion

In this study, seven biomarkers reported to be related to lymph node metastasis in the literature were examined, and only CD44v6 was found to be an independent predictive marker for lymph node metastasis. Based on the multivariable analyses, we developed nomograms predicting lymph node metastasis in early gastric cancer, and the predictability of lymph node metastasis was improved by adding CD44v6 to conventional clinicopathological factors. Additionally, functional study revealed that CD44v6 played a role in cell proliferation, migration and invasion.

In previous studies, the seven biomarkers were proved to be associated to lymph node metastasis in gastric cancer. [7, 8, 16–21] However, in this study, E-cadherin, α1 catenin, p53, EZH2 had no relationship with lymph node metastasis, and Annexin II and PRL-3 had little discrimination in the immunohistochemistry. CD44v6 was the only significant risk factor for lymph node metastasis, and its OR was very high similar to depth of invasion.

CD44 is a highly glycosylated transmembrane protein and is expressed in a variety of epithelial and mesenchymal cells as well as tumor cells. [22] CD44 isoforms are generated by alternative splicing of at least 12 exons and are correlated with regulating tumor invasion, progression, and metastasis in various malignancies including gastric cancer. [23–25] In particular, high expression of CD44v6 is significantly correlated with lymphatic invasion and lymph node metastasis in gastric cancer, and associated with worse overall survival of gastric patients. [16, 26–30] This study also showed a relationship between CD44v6 and lymph node metastasis in early gastric cancer.

The mechanisms of CD44v were evaluated in several different cancer cell lines, which were associated with arsenic-induced neoplasmatic transformation, invadopodia formation, and AKT-mediated pathway. [31–34] However, little is known about the mechanism of CD44v6 in gastric cancer. This study just identified oncogenic function of CD44v6 in cell proliferation, migration and invasion in gastric cancer cell lines, and further study is needed to identify the specific mechanism of CD44v6 in gastric cancer.

Although this study developed a new biomarker prediction model by adding CD44v6 to the conventional clinicopathological factors, the increases of AUCs was not remarkable. However, it is well-known that a significant increase in AUC is very difficult without a high degree of association of the new marker. [35, 36] Instead, this study showed that NRI had significant value (*p* < 0.05), which indicates significant improvement in the model performance by adding CD44v6 to conventional model.

The nomograms without LBVI can easily be used in decision making for treatment plan by calculating the probability of lymph node metastasis. A physician can inform a patient of the calculated probability of lymph node metastasis and suggest a suitable treatment modality considering patient’s comorbidity. Moreover, the nomogram with LBVI can be used after ESD, and a patient can understand why additional surgery is needed based on the calculated probability of lymph node metastasis.

One of limitations of this study is the small sample size, and 336 early gastric cancer tissues may be insufficient to develop prediction models. Second, external validation was not performed due to practical problems, and internal validation by boot-strapping was done. Third, only seven available markers were examined in this study, and the nomograms did not include all previously reported biomarkers related with lymph node metastasis.

In conclusion, CD44v6 is an independent predictor for lymph node metastasis in early gastric cancer. The probability of lymph node metastasis can be calculated using the developed nomograms, and the nomograms could be helpful in determining treatment plans.

## Supporting Information

S1 Table The clinicopatholgical characteristics according to the expression of CD44v6. (DOCX)

S1 File Results of immunohistochemistry. (XLSX)

S2 File Results of statistical analysis. (XLSX)

S3 File Results of cell line experiment. (XLSX)
