# Supplementary material for: Nomogram Incorporating CD44v6 and Clinicopathological Factors to Predict Lymph Node Metastasis for Early Gastric Cancer
Source: PLoS One. 2016 Aug 2;11(8):e0159424. doi: 10.1371/journal.pone.0159424 (PMC4970798; doi:10.1371/journal.pone.0159424)
Supplement: S1 Table — (DOCX) [file pone.0159424.s001.docx]

S1 Table. The clinicopatholgical characteristics according to the expression of CD44v6

| Characteristics | Subgroup | Total | CD44v6 low (%) | CD44v6 high (%) | *p*-value |
| --- | --- | --- | --- | --- | --- |
|  |  | ( n=336) | ( n=305 ) | ( n=31 ) |  |
| Age (mean±SD) |  | 336 | 58.3 ± 10.6 | 58.6 ± 12.6 | 0.934 |
| Sex | Male | 211 | 192 (63.0) | 19 (61.3) | 0.99 |
|  | Female | 125 | 113 (37.0) | 12 (38.7) |  |
| Size (mean±SD) |  | 336 | 3.2 ± 1.9 | 3.5 ± 1.9 | 0.273 |
| Histological type | Differentiated | 81 | 188 (61.6) | 24 (77.4) | 0.117 |
|  | Undifferentiated | 60 | 117 (38.4) | 7 (22.6) |  |
| LBVI | Not identified | 140 | 281 (92.1) | 25 (80.6) | 0.045 |
|  | Present | 1 | 24 (7.9) | 6 (19.4) |  |
| Ulceration* | Absent | 140 | 290 (95.1) | 30 (96.8) | 0.999 |
|  | Present | 1 | 15 (4.9) | 1 (3.2) |  |
| Depth of invasion | Mucosa | 195 | 180 (59.0) | 15 (48.4) | 0.34 |
|  | Submucosa | 141 | 125 (41.0) | 16 (51.6) |  |
| LN metastasis | negative | 312 | 288 (94.4) | 24 (77.4) | 0.003 |
|  | positive | 24 | 17 (5.6) | 7 (22.6) |  |
| E-cadherin | Not reduced | 115 | 257 (84.3) | 30 (96.8) | 0.063 |
|  | Reduced | 26 | 48 (15.7) | 1 (3.2) |  |
| α1 catenin | Not reduced | 93 | 225 (73.8) | 25 (80.6) | 0.519 |
|  | Reduced | 48 | 80 (26.2) | 6 (19.4) |  |
| p53 | Low | 84 | 208 (68.2) | 14 (45.2) | 0.016 |
|  | High | 57 | 97 (31.8) | 17 (54.8) |  |
| EZH | Not reduced | 112 | 241 (79.0) | 30 (96.8) | 0.029 |
|  | Reduced | 29 | 64 (21.0) | 1 (3.2) |  |
